# Proposal of a Lab Bench for the Unobtrusive Monitoring of the Bladder Fullness with Bioimpedance Measurements

**DOI:** 10.3390/s20143980

**Published:** 2020-07-17

**Authors:** Valentin Gaubert, Hayriye Gidik, Vladan Koncar

**Affiliations:** 1 GEnie et Matériaux TEXtiles (GEMTEX) Laboratory, École Nationale Supérieure des Arts et Industries Textiles (ENSAIT), F-59100 Roubaix, France; hayriye.gidik@yncrea.fr (H.G.); vladan.koncar@ensait.fr (V.K.); 2 Hautes Etudes Ingénieur (HEI)—YNCREA, University of Lille, F-59650 Villeneuve d’Ascq, France

**Keywords:** bioimpedance, pelvic phantom, bladder volume monitoring, urinary incontinence

## Abstract

(1) Background: millions of people, from children to the elderly, suffer from bladder dysfunctions all over the world. Monitoring bladder fullness with appropriate miniaturized textile devices can improve, significantly, their daily life quality, or even cure them. Amongst the existing bladder sensing technologies, bioimpedance spectroscopy seems to be the most appropriate one to be integrated into textiles. (2) Methods: to assess the feasibility of monitoring the bladder fullness with textile-based bioimpedance spectroscopy; an innovative lab-bench has been designed and fabricated. As a step towards obtaining a more realistic pelvic phantom, ex vivo pig’s bladder and skin were used. The electrical properties of the fabricated pelvic phantom have been compared to those of two individuals with tetrapolar impedance measurements. The measurements’ reproducibility on the lab bench has been evaluated and discussed. Moreover, its suitability for the continuous monitoring of the bladder filling has been investigated. (3) Results: although the pelvic phantom failed in reproducing the frequency-dependent electrical properties of human tissues, it was found to be suitable at 5 kHz to record bladder volume change. The resistance variations recorded are proportional to the conductivity of the liquid filling the bladder. A 350 mL filling with artificial urine corresponds to a decrease in resistance of 7.2%, which was found to be in the same range as in humans. (4) Conclusions: based on that resistance variation; the instantaneous bladder fullness can be extrapolated. The presented lab-bench will be used to evaluate the ability of textiles electrodes to unobtrusively monitor the bladder volume.

## 1. Introduction

Bladder control is regulated by complex neural mechanisms that are still not fully understood [1]. This absence of bladder sensation can result in either (i) urinary retention, which can lead to severe complications, such as urinary tract infections [2], or (ii) urinary incontinence. These conditions can impact deeply the patient’s quality of life [3] if bladder fullness is not monitored in real-time by a non-invasive device that enables the patient to carry out everyday activities. 

To this purpose, and thanks to the progress in electronics and wireless communications, the field of Ambulatory Urodynamic Monitoring (AUM), whose methods are used to rely on traditional catheters, is now being revolutionized to evolve towards novel Telemetric AUM (TAUM) [4]. The non-invasive methods to sense bladder fullness can be divided into three main techniques: ultrasonography [5], near-infrared spectroscopy (NIRS) [6], and bioimpedance [7]. 

In addition, to be non-invasive, one of these techniques could be made wearable if seamlessly integrated into an undergarment; hence, enabling unobtrusive continuous and long-term monitoring of the bladder, as it was done for cardiac [8] or brain [9] activities. Bioimpedance sensing seems to be the most suitable one to be integrated into textile clothing. Indeed although recent advances in material science enable the realization of high-performance piezoelectric systems in soft and flexible formats [10], the ultrasonic transducer can be integrated on polydimethylsiloxane (PDMS) [11] substrate, but not on textile fibers directly. Near-infrared spectroscopy, which relies on the different absorption properties of human tissues and water, is promising, but glass fiber cannot be so much bent when seamless knitting required [12]. 

Bioimpedance or biological impedance is defined as the ability of biological tissue to impede electric current [13]. It utilizes the difference of conductivity of the urine compared to the surrounding tissues and has already been used to determine the bladder fullness. Early work on dog bladder volume sensing with impedance measurements began in the 1970s [14]. Doyle et al. [15] and Abbey et al. [16] reported the first trials on humans in 1975 and 1983. More recently, wearable noninvasive bladder urinary volume monitoring devices, which were specifically designed [17] or taken off-the-shelf from electronic suppliers [18], have proven promising results. Nonetheless, to the best of our knowledge, no attempt on merging such electronic monitoring devices into textile underwear has been reported yet. However, some textile bioimpedance sensors have been developed for Body Composition Analysis (BCA) [19] or Bioelectrical Impedance Analysis (BIA) [20].

To evaluate the feasibility of using smart underwear to monitor the bladder urinary volume, a lab bench, including a realistic pelvic phantom, was needed. This study aims at designing and fabricating such pelvic phantom and comparing it to the human body; hence, its suitability can be discussed. More specifically, the phantom has been designed to reproduce enuretic child bodies—who are mostly 5 to 10 years old (75%) [21]. 

Most of the anthropomorphic pelvic phantoms presented in the literature have been fabricated for radiation therapy dosimetry [22,23,24,25,26,27,28,29]. Hence, the phantom materials used were selected for their radiological properties, such as Hounsfield unit (HU) [22,30], rather than their electrical properties. As a result, their structures mainly consist of non-electrically conductive materials, such as polymethylmethacrylate (PMMA) [22,31]. Consequently, these phantoms are not suitable for this study.

Few phantoms for electrical impedance have been reported. They reproduce head [32,33] breast [34,35,36] or thorax [37].

Only one adult pelvic phantom for impedance measurements can be found in the literature [38]. It was only designed to assess predetermined bladder volumes of 40, 60, 100, 160, 240, and 400 mL by inserting conductive polyurethane spheres in the phantom background. The present study aims at going a step further by fabricating a phantom, enabling real-time monitoring of the effective bladder filling as it takes place inside the human body. Furthermore, the structure of the phantom proposed by Dunne et al. [38] is not adapted to wear textile underwear, as its shape does not have legs. Moreover, its material may not be strong enough to withstand the mechanical constraints exerted by the elastane knitted in the underwear; hence, the need to fabricate another pelvic phantom. In the Material and Methods Section, the design of each element of the pelvic phantom is detailed; then the whole lab-bench is explained. In the Results Section, experimental measurements of the resistive and reactive parts, acquired on the lab bench, are presented. More specifically, the results obtained on the lab bench have been confronted with those obtained on humans. Moreover, the recordings of the bladder filling with solutions of different conductivities are shown. Finally, these results are discussed, regarding the fidelity of the presented phantom compared to humans, and the measurements’ reproducibility. Moreover, the limitations of the designed lab bench are raised to define the main challenges for future work. 

## 2. Materials and Methods

### 2.1. Lab-bench

#### 2.1.1. Manikin

To have a realistic shape, a commercial pelvic boy manikin (RETIF, Villeneuve-Loubet, France) has been purchased from RETIF (France). Moreover, the structure needs to be rigid enough to hold the textile underwear, composed of 10% elastane (FILIX, Creney, France), which tends to spring back to its original length when extended. As the manikin is made of plastic, which is not electrically conductive, it has been drilled at four different locations, where the electrodes should be placed. Hence, they will be in direct contact with the conductive internal background so that impedance measurements can be realized. 

**Figure 1:**
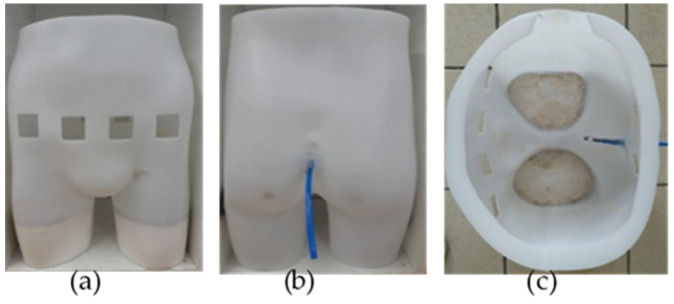
Manikin seen from different perspectives: (**a**) front view (left), showing the four holes corresponding to the electrodes’ locations; (**b**) back view (middle), showing the emptying pipe; (**c**) top view (right), showing the manikin’s interior before being filled with the agar background and pig’s bladder.

Multiple views of the manikin are given in Figure 1. The top of the manikin was opened to enable its filling with the phantom background and the artificial urine thereafter. The bottom of each leg was hermetically sealed to retain the background. Few centimeters of plaster were placed in the legs to stabilize the manikin and reduce the background volume to be filled with. 

#### 2.1.2. Internal Background

Many tissue-mimicking phantom materials can be found in the literature, such as agar [39], polyvinyl alcohol, gelatin [40], polysaccharide gels TX-150, carrageenan, polyacrylamide, sodium alginate [41], xanthan gum [41], polyacrylic acids, such as Carbomer-980 [41], and superabsorbent polymers [41]. Electrical conductivity is the main criterion for selecting the background material, which should be adjustable to match the human one in the pelvic cavity. Since the manikin has been drilled, the background material must be solid, otherwise it would spill out from the holes, making any measurements impossible. Furthermore, since there was quite a large volume (approximately 15 L) to be filled with the internal background, the cost of the material has also been taken into account. Conductive fillers, such as carbon nanotubes or silver powder, were once considered, but discarded regarding their high prices. According to Kandadai et al. [42], the electrical conductivity of the agar phantom can be adjusted by doping it with sodium chloride (NaCl). Hence, agarose based tissue substitutes have been widely used in the literature to mimic several organs (brain, thyroid gland, breast [35]), for many applications, such as ultrasound imaging. In addition to being non-expensive, agar is not hazardous and easy to prepare. 

However, it is not stable for long periods of time, as it tends to dry out rapidly (within two days in the open air), and is conducive to the growth of bacteria. To cope with the proliferation of bacteria, few drops of an antibacterial and antifungal conservative (Cosgard, Aroma-Zone, Paris, France) were added to the preparation, and the manikin was stored in an industrial refrigerator. As a result, the phantom’s lifetime has been extended to up to two weeks. 

**Figure 2:**
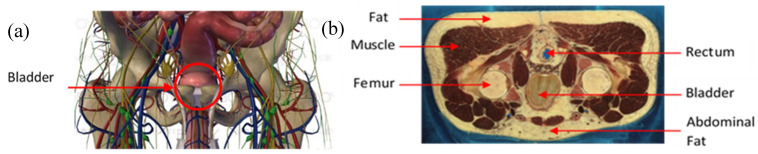
(**a**) Anatomy of the pelvic cavity showing the bladder’s location; (**b**) axial computer tomography (CT) scan of the pelvic cavity presenting the nature and location of the tissues.

The agar powder (Biocean, Roscoff, France) was dissolved in deionized water and the electrical conductivity of the preparation was determined by using sodium chloride, NaCl, (alimentary salt, les Salins du Midi, Aigues-Mortes, France). The salt was added to the preparation in a precise quantity to match the theoretical conductivity of the human pelvic cavity. Figure 2a shows an online three-dimensional (3D) anatomical model of this pelvic region. Figure 2b is a male computer tomography scan (CT scan) extracted from the Visible Human Project [43]. Table 1 gives the electrical conductivities of the main elements that can be distinguished in the pelvic cavity in Figure 2a,b. These values have been taken from two online databases, [44,45], at the frequency of 5 kHz. An agar phantom, with an average conductivity of all the mentioned elements in Table 1, was prepared. Thus, the designed phantom presented in this study does not match perfectly the human body, regarding the complex volume distribution of the electrical conductivities. Nonetheless, it is acceptable to realize proof-of-concept or pre-clinical measurements.

Its overall conductivity was determined by a weighted average of the volume occupied by muscles, fat, bones (pelvic, coccyx, femur), and pelvic cavity organs, such as the rectum. The volumes of each tissue were approximated by interpreting all the male computer tomography scans from the Visible Human Project [43], on which the bladder can be found. The following estimation has been taken to calculate the average conductivity of the background: muscle (50%), fat (30%), bones (15%), and rectum (5%). The conductivity of each element is given in Table 1.

As bone marrow is a combination of hematopoietic red marrow and yellow fatty marrow [46], whose conductivities are very different, they have been separated for the calculation. Since their proportions vary during life [47]: at birth, the bone marrow mostly contains hematopoietic elements, which are progressively replaced by fat until adulthood, when the bone marrow fat represents 70% of the bone marrow volume [48]. For 6–12 year old kids, the red marrow represents 50 to 70% [47] of the bone marrow, so 2/3 of red marrow was taken into account for the calculation; hence, the respective percentage given in Table 1. 

The resulting weighted conductivity of the internal background is approximately 0.2 S/m, which is interestingly close to the conductivity of the bladder wall. Thus, at first, urine was thought to be put directly in the hole inside the internal background. However, although the agar has been prepared to be compact, it is not completely impervious so far, especially to ions. Therefore, the ions suspended in the salted water, mimicking the urine, tended to migrate and penetrate the agar background, increasing irremediably its conductivity. That is why a proper hermetic bladder was needed to contain the artificial urine to be filled. Such bladder has been placed right into that hole, surrounded with few milliliters of salted deionized water, with a conductivity of 0.2 S/m, like the background, to connect the bladder with the agar hole walls; thus, properly ensuring the electrical conductivity. 

**Figure 3:**
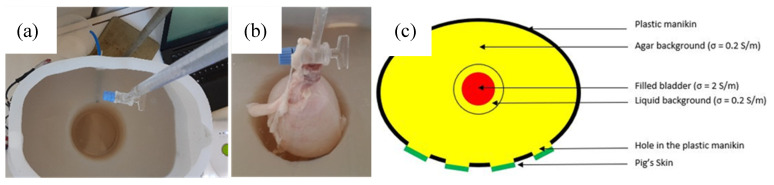
(**a**) Agar background with the hole for the bladder, which includes an emptying pipe; (**b**) expanded pig’s bladder, inserted in the agar’s hole, being filled with the artificial urine; (**c**) layout of the phantom, presenting the different layers with their electrical conductivities (top view).

However, it was found that the level of water inside the agar hole must be kept constant. Otherwise, the recorded impedance variations reflect the increase of the water level inside the hole, due to the bladder expansion, but not the artificial urine put inside the bladder. It has been noticed that whatever the conductivity of the liquid shed inside the bladder, the recorded impedance magnitudes and variations were very similar. Still, the only way to keep the level of water constant inside the hole is to evacuate continuously the background solution in excess due to bladder expansion. Consequently, a small emptying pipe has been installed at the back of the manikin (see Figure 3). Although this is not what happens exactly in the human body, this pipe enables to work with a precise constant volume. As a result, the bladder volume, filled with artificial urine, was the only changing parameter inside the pelvic phantom. Therefore, being able to detect this bladder increase with bioimpedance measurement is the aim of this study. 

The quantity of salt (NaCl) needed to be added into the agar phantom was calculated by using Equations (1) and (2).

σ = λCl^−^·[Cl^−^] + λNa^+^·[Na^+^] = (λCl^−^ + λNa^+^)·x (1)

where σ is the conductivity of agar (0.2 S·m^−1^), λCl^−^ is the molar conductivity of Cl^−^ (7.63 × 10^−3^ S·m^2^·mol^−1^), λNa^+^ is the molar conductivity of Na^+^ (5.01 × 10^−3^ S·m^2^·mol^−1^) and x is the molar concentration of NaCl (mol·m^−3^).

The molar concentration of salt needed (called x) is 15.9 mol·m^−3^.

y = M(NaCl)·x(2)

y is the weight of NaCl (g·m^−3^), M(NaCl) is the molar mass of NaCl (58.44 g·mol^−1^) and x is the the molar concentration of NaCl (15.9 mol·m^−3^). The weight of salt needed (called y) is 925 g·m^−3^ or 0.925 g·L^−1^.

To sum up, the internal background (see Figure 3a) was prepared with the following recipe:

15 L of deionized water

150 g of agar powder (10 g·L^−1^)

13.9 g of alimentary salt (0.925 g·L^−1^) 

50 drops of conservative. 

The resulting conductivity, measured with a conductometer (sension + EC7, Hach) at 5 kHz, was 0.21 ± 0.2 S/m. 

#### 2.1.3. Skin

**Figure 4:**
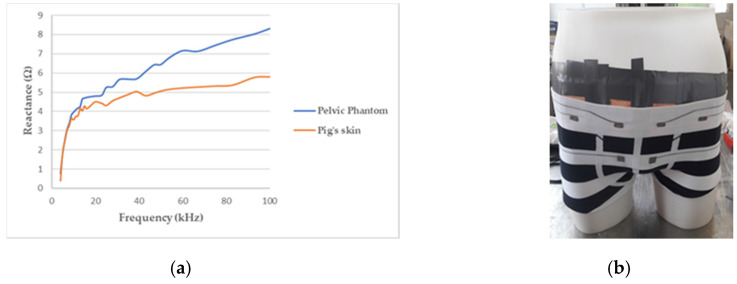
(**a**) Reactance of the pelvic phantom compared to the pig skin; (**b**) manikin wearing a boxer underwear incorporating flexible electrodes for bioimpedance measurement.

As the skin, particularly the outermost layer called the stratum corneum, and the subcutaneous fat, are much less conductive than the tissues below (see Table 1), another layer, above the internal background was needed to obtain a realistic phantom. Therefore, pig skin has been stuck on the manikin in direct contact with the agar background. Indeed, the pig skin anatomy, biological composition, and mechanical properties, are very close to human skin [49]. It accounts for the major part of the phantom’s reactive component, as illustrated by Figure 4a.

#### 2.1.4. Bladder

The fabrication of a functional bladder replica was found to be the most difficult. Indeed, the developed test bench was designed to realize dynamical measurements, i.e., with a continuous filling of the bladder compared to the phantom developed by Dunne et al. [38]. To this purpose, the bladder mimicking material must be not only electrically conductive, but also has very specific mechanical properties. It needs to be elastic to expand with the filling and resistant to bear the weight of the filler. In the literature, only two complex bladder phantoms were found for medical applications [22,50]. To go further into building a more realistic pelvic phantom, a pig’s bladder was used in addition to pig skin. This pig’s bladder was provided immediately post-mortem by an abattoir to realize ex vivo measurements on the developed test bench. A real pig’s bladder has already been used by Fong et al. [6] with the intestines.

Filling the manikin with pig viscera, dipped into agar, to fabricate an even more realistic manikin has also been considered once. Indeed, pig tissues could have improved the permittivity of the background, making it closer to the human one. However, the phantom lifetime would have been too short, and some hazardous bacteria could have developed inside. Figure 3c shows an overall layout of the complete phantom.

#### 2.1.5. Conductive Filler

The bladder was then filled with artificial urine at the desired flow rate with a precise burette. Some detailed recipes of synthetic urine can be found in the literature [51]. However, as only its conductivity matters for these bioimpedance measurements, only alimentary salt was added to deionized water to adjust the conductivity of the filling solution. Since the conductivity of urine depends on several factors such as individuals, diet, or time of urination, many figures, varying from 1.75 S/m [44] to 2.15 S/m [52] can be found in the literature for its reference value. 

The bladder was filled with a conductive solution at 2 S/m, which was all the more practical than it was exactly 10 times more than the internal background. According to the previous calculation, the solution was prepared with 9.25 g of alimentary salt per liter of deionized water. The measured conductivity of the filling solution was also slightly higher than expected (2.15 S/m). Then, the solution was shed almost constantly with a burette at a flow rate of approximately 1 mL per second.

In addition, five different solutions were prepared to record different magnitudes of decline and investigate an eventual correlation between the conductivity and the decline coefficient trend. Solution 1 consists of demineralized water, which is not electrically conductive. Solution 2 is the background solution, in which the bladder is suspended. Solution 3 is supposed to be low concentrated urine (1 S/m), solution 4 represents the average urine conductivity (2 S/m), and solution 5 is an extremely high concentrated urine (6 S/m). The recipe and conductivities of those solutions are given in Table 2. 

### 2.2. Bioimpedance Measurement

Electrical impedance is the capacity of a material to impede the flow of an electrical current that is applied to it. More specifically, when the material is a biological one, as the human body, the term bioimpedance is used. Nonetheless, there is a fundamental difference between materials, such as metals and biological tissues regarding the nature of the electrical charges, which are respectively electrons and ions. Bioimpedance measurements consist in applying an electrical current of low amplitude, by two electrodes in direct contact with the skin, through the human body, and measure the corresponding voltage drop and phase shift with the same (or two other) electrodes. Impedance can be then calculated by applying Ohm’s law, and phase shift can also be obtained. 

The human body is composed of aqueous tissues, fat, and bones. Whereas body fat and bones have poor electrical conductivity, the aqueous tissues, rich in electrolytes, are electrical conductors. The specific conductivity of different tissues has been studied by researchers such as Gabriel et al. [53] at different frequencies and the values can be accessed on an online database Indeed, the human body, more precisely biological tissues, shows frequency-dependent behavior, as its water content consists of two different parts [54]: the intracellular fluid contained inside the cells and the extracellular fluid, in which cells are suspended. Those two fluids are separated by a cell membrane, which is a fatty layer inducing a capacitance. Like a capacitor, it behaves like an open circuit at low frequencies as charges accumulate on the membrane, but the current cannot pass through it. Hence, it flows mostly through the extracellular fluid. When the frequency increases, the charge–discharge process is getting so fast that the cell membrane effect is very small and current flow through the cell [55].

As a result, the overall response of the biological tissues to an alternating electrical signal applied to it produces a complex bioelectrical impedance depending on tissue composition as well as the frequency of the applied alternating current [56]. The specific frequency at which the current begins to pass through the cells is called the characteristic frequency [57], and varies around 50 kHz according to the individual. This 50 kHz frequency has been historically used to determine body composition, i.e., evaluating the proportions of the different body compartments: fat mass, free fat mass, and water [58]. Single Frequency Bioimpedance Analysis (SF-BIA) at 50 kHz has even become one standard among the bioimpedance measurement techniques. As a result, most of the (few) studies found on the evaluation of the bladder level, thanks to bioimpedance measurements, use this 50 kHz frequency [59,60,61].

However, as urine is specifically an extracellular fluid, it seems more logical to investigate only the extracellular fluid rather than adding the intracellular ones. The frequency cannot be too low though, otherwise the current will not pass through the subcutaneous fat; thus, a working frequency of 5 kHz has been applied. Regarding the difficulties to cross the fatty layer on real subjects, this value can be increased thereafter. A tetrapolar electrode configuration, consisting of two current injecting electrodes and two voltage-sensing ones, has been preferred to the two-electrode-method to overcome the electrode-skin impedance [62]. The voltage sensing electrodes were placed inside the current injecting ones. Conventional gelelectrodes (Asept InMed, Quint-Fonsegrives, France), used for electrocardiography, were placed on the skin at four different locations on the lower abdomen. Textile electrodes, characterized in a previous study [63], will be used in future work. The ECG electrodes were connected to a multi-frequency impedance analyzer (Zscan, Bioparhom, Challes Les Eaux, France), which was linked to a computer displaying the results in real-time on the dedicated interface.

**Figure 5:**
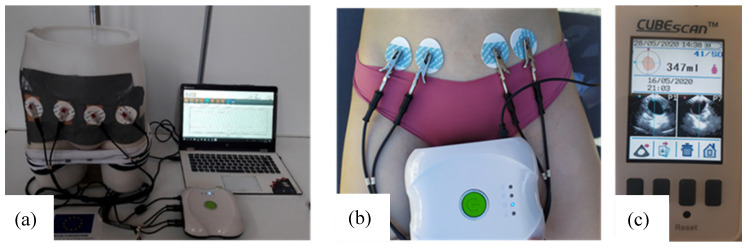
(**a**) The whole test bench including the pelvic phantom, the measuring device and a computer; (**b**) tetrapolar bioimpedance measurements realized on individual 2; (**c**) bladder volume before voiding recorded by the bladder scan (screenshot).

The whole test bench is shown in Figure 5a. It consists of the designed pelvic phantom, inside which the bladder is precisely filled with salted water (representing urine) by a burette. Simultaneously, the impedance meter records in real-time the variations of impedance at the surface of the phantom’s skin with four electrodes. The manikin is suitable for wearing textile boxer underwear (see Figure 4b). A further study will present the integration of impedance electrodes into that boxer and the results obtained on the presented lab bench. 

However, its suitability to assess the bladder fullness has to be investigated before doing those further measurements. Therefore, several experimental measurements have been realized. Three bladder fillings with artificial urine and bladder removals have been recorded to evaluate the reproducibility on the lab bench. Then the bladder was filled with the liquids of different electrical conductivities, given Table 2, to ensure the reliability of the lab bench. It seems important to be able to record—not only resistance decreases—but also increases and stagnation to prove it. 

Moreover, the pelvic phantom fabricated has been compared to the human pelvis to evaluate its fidelity. Two volunteers, one male, and one female took part in this experiment. All subjects gave their informed consent for inclusion before they participated in the study. The study was conducted in accordance with the Declaration of Helsinki, and the protocol was approved by the Ethics Committee of Gemtex laboratory. None of the subjects had any history of urological or neurological diseases. The experiment was performed in a room with a temperature set to 25 °C and relative humidity between 60% and 70%. Before the experiment, subjects were required to void their bladder. Then they drank 500 mL of mineral water and lay on a bed. Four ECG medical gel electrodes (WhiteSensor WS, Ambu, Ballerup, Denmark) were placed on their abdomen as on the manikin. Bioimpedance measurements were recorded by the Zscan (70 µA, 5 kHz). One measurement was done at the very beginning of the experiment when the bladder can be considered empty. Then the bladder volume was evaluated by a bladder scan (Biocon-900, MCube Technology, Seoul, Korea) every 15 min until 350 mL was reached. At that moment, bioimpedance was recorded, then the individuals were asked to void. Another measurement was done just after the voiding to calculate the bioimpedance variation corresponding to a bladder filling of 350 mL. 

## 3. Results

**Figure 6:**
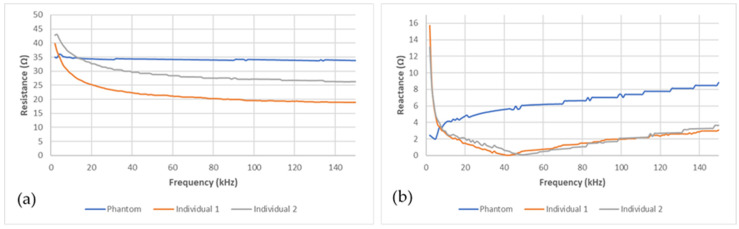
Comparison between the phantom’s (blue line) (**a**) resistance and (**b**) reactance and the human ones (grey and red lines).

The bioimpedance measurement device was able to acquire data stably after the electrodes, stuck on the pelvic phantom, were connected. Figure 6a,b present, respectively, the electrical resistance and reactance recorded on the pelvic phantom compared to the human ones in the frequency range (1–150 kHz). Whereas resistance graphs for the two individuals show the same trend, as frequency increases the resistance decrease, the phantom one is not frequency-dependent behaving as a pure resistance. Moreover, the reactance recorded for the two individuals is very similar; a sharp decrease at very low frequencies followed by a smoother one until reaching the characteristic frequency (around 40 kHz), then it increases. Unlike them, the phantom one keeps increasing without showing any characteristic frequency in this frequency range. Those findings are discussed in the following section. Moreover, it should be noted that the reactance magnitude at low frequencies is smaller than the resistance one. Consequently, the reactance squared is neglectable compared to the resistance squared. Furthermore, no meaningful correlations were found between the reactance variations and the bladder volume during the bladder filling. As a result, only the resistance variations were taken into account and presented in this study. 

**Figure 7:**
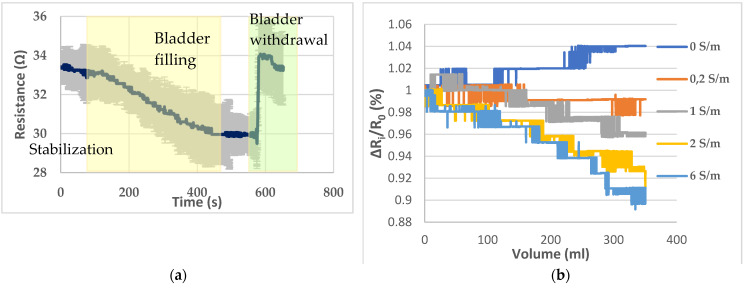
(**a**) Bladder filling and withdrawal recording; (**b**) resistance variations recorded with the different solutions presented by Table 2.

The electrodes, placed on the pig skin, were able to detect the bladder filling with artificial urine (solution 4) that takes place inside the pelvic phantom. This filling has been reproduced three times in a row. The mean graph, with standard deviation, resulting from the monitoring of the bladder filling and withdrawal is given by Figure 7a. The average coefficient of variation is 3.86% between the three trials with a minimum of 1.11% and maximum of 5.97%. A stabilization period of 100 s has been observed before the filling. Hence, the resistance drop due to the bladder’s filling can be clearly distinguished from a weak natural drift. In addition, the bladder was removed 75 s after the end of the filling to assess that the resistance magnitude change was directly correlated with the filled bladder. 

Then, to assess the ability to detect liquids of different electrical conductivities and distinguish them, the five solutions presented in Table 2 have been poured successively inside the bladder. Figure 7b presents the resistance variations corresponding to the filling with the different solutions. A ratio ΔR_i_/R_0_, where ΔR_i_ (Ω) = R_v_ − R_0_ with R_v_ corresponding to the resistance detected by the sensor when the volume V has been poured, and R_0_ is the initial resistance has been calculated to standardize the variations and compare them. Indeed, as the bladder needs to be withdrawn to be emptied after each filling, there was a little change in the initial value of resistance measured. 

**Figure 8:**
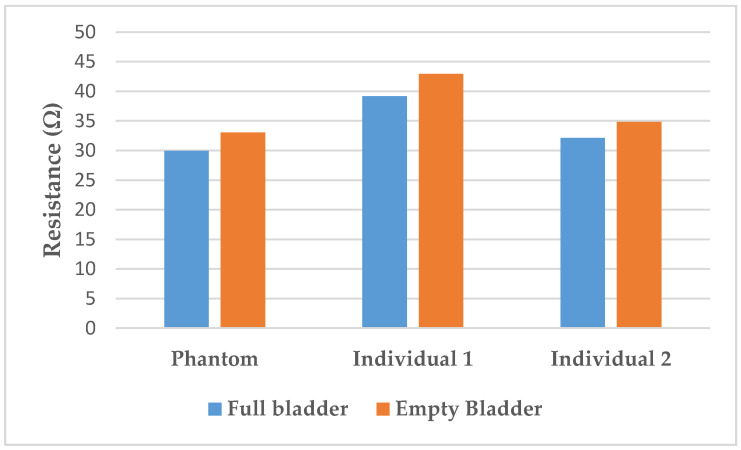
Resistance variation between full and empty bladder on the phantom compared to on the individuals.

The resistance variation corresponding to the 350 mL filling of the bladder with artificial urine on the lab bench is 9.3 ± 0.3%. In the human subjects, variations of 9.7% for individual 1 and 8.5% for individual 2 were calculated. Those variations are illustrated in Figure 8.

## 4. Discussion

Regarding Figure 6a,b, the fabricated phantom does not mimic the frequency-dependent electrical properties of the human body. Indeed, resistance does not vary with frequency. Moreover, the reactance does not decrease until the characteristic frequency is reached. Not simulating the permittivity of tissue is one of the weaknesses of the agar/saline phantoms developed. Indeed, this is the main weakness of agar/saline phantoms. The addition of biological materials with cellular structures, such as cucumber [53], can overcome this shortcoming. However, they have not been used because of their very short lifetime and the lack of reproducibility of their electrical properties. Moreover, inorganic materials, such as graphite [54] or polymer microbeads could be added to simulate phantom permittivity. Nonetheless, those microbeads need a proper stirring to obtain a homogeneous phantom. However, because of the shape of our manikin, stirring has been found difficult to maintain inside the manikin, once agar is poured, especially in the two legs. As a result, the phantom is not suitable for multi-frequency bioimpedance analysis. However, at 5 kHz, both the resistance and reactance of the phantom are in the same range as humans’ one. 

Although the measurements seem relatively stable, according to Figure 7a, they were done successively. Nonetheless, the agar background resistance increases irremediably as it dries out. Furthermore, the pig skin has to be changed every 3 h and the electrical properties vary from animal to animal. For those reasons, the reproducibility of this pelvic phantom is limited. 

The presented pelvic phantom is suitable to monitor the bladder filling, regarding Figure 7b. With deionized water, the measured resistance tends to increase as the deionized water is not as conductive as the background. As expected, practically no resistance variations were observed when the filler was the background solution. Then resistance drops were proportional to the conductivity of the filler, i.e., the more conductive the larger the drop. For solutions 3 and 4, the decline coefficient trend seems proportional to the electrical conductivity regarding the resistance decrease. However, it is not the case for solutions 4 and 5, whose decline is relatively close, whereas solution 5 is three times more concentrated than solution 4.

Furthermore, the resistance variation of 9.3% with the filling is relatively close to those measured on humans (respectively, 9.7% for individual 1, and 8.5% were calculated). Those results are in accordance with the findings of Shida et al. who reported a resistance variation range from 5 to 12% with two dominant rates of 8 and 9%. 

**Figure 9:**
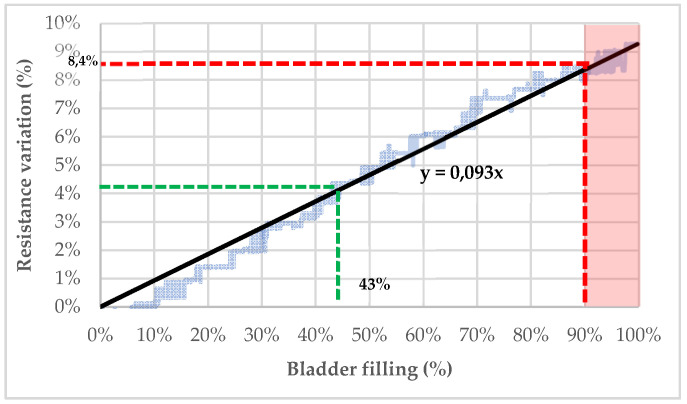
Bladder’s filling estimation based on resistance variation recorded by the electrodes.

As shown in Figure 9, the content of the pig’s bladder can be theoretically determined by resistance measurements. The bladder was filled up to 350 mL, which corresponds approximately to the theoretical bladder capacity of an 11-year-old child [64].

Considering 350 mL as the maximal capacity of a child’s bladder (100% filled), it is possible to extrapolate the filling percentage of the bladder from the variation of resistance (see Figure 9). Indeed, with a linear regression based on the trendline of the resistance decrease following the bladder filling with artificial urine (C = 2 S/m), the bladder’s fullness can be determined. For example, a resistance variation of 4% seems to indicate that the bladder is filled at 43%. To prevent bedwetting, a threshold after which micturition is very likely to occur can be established. If this threshold is settled at 90% of the bladder capacity, the child should be woken up when the detected resistance variation exceeds 8.4%. 

All things considered, the fabricated pelvic phantom seems to be suitable to monitor the bladder volume. However, it should be kept in mind that the developed lab-bench presents some limitations. On the one hand, human tissues are far more complex than the designed phantom. Not only because human cells have a frequency-dependent behavior, which was not taken into account, but also, because those cells, and organs at a larger scale, are living tissues so in perpetual change. Indeed, whereas the bladder filling is the only phenomenon that occurs inside the designed pelvic phantom, many others take place simultaneously inside the human body. These physiological phenomena are associated with the digestive, cardiovascular, or endocrine systems. Consequently, the impedance drop, which can be identified on the developed lab-bench, can be drowned among other phenomena when monitoring real patients. On the other hand, whereas the designed manikin is standing still, sleeping humans lay horizontally and are prone to move during the night. This can affect both the contact impedance between the skin and the electrodes, but also the bladder and all tissue position. Consequently, the volume of urine detected by the bioimpedance sensors can be impacted. 

For all those reasons, further human tests would be needed to calibrate a bladder volume-monitoring device, as many parameters, such as skin impedance, bladder volume and position, urine conductivity, vary from one individual to another. Such clinical trials should investigate the inter-variability and intra-variability of urine conductivity to determine if such a monitoring device should implement machine-learning techniques to adapt specifically to its wearer. Moreover, as several configurations of the four electrodes can be considered, the best one, according to the patient’s position (supine, sitting, standing), can be evaluated. Meanwhile, the impact of organ position on the measured impedance can be estimated. Indeed, because of gravity, the precise bladder location, as well as the surrounding tissues, varies when the individual changes its position from lying to standing, particularly the relative position of the electrodes comparing to the bladder position. Compared to the phantom presented in the literature, the anatomy of the pelvic cavity could be further improved by 3D printed the pelvic and femoral bones. The conductivity of printable filaments can be tailored by mixing acrylonitrile butadiene styrene (ABS) and carbon black (CB) particles [65]. Moreover, the manikin structure can be 3D printed with a material reproducing the skin conductivity. It would be even better to 3D print a manikin with a soft material; hence, the phantom modularity and flexibility would be significantly enhanced. Indeed, multiple electrode locations can be tested to determine the best configuration. Moreover, different sizes of phantom could be easily printed to reproduce specific morphologies (child/adult, male/female). It would enhance the reproducibility of the measurement, as the 3D printed material conductivity is stable over time compared to the pig skin that dries out and must be changed every three hours. 

## 5. Conclusions

A realistic pelvic phantom, incorporating ex vivo tissues such as pig’s bladder and skin, has been designed and fabricated. The main criteria taken into account for designing such a phantom are: (1) dielectric properties similar to human tissues, (2) homogeneity of those properties in the phantom or desired specific areas, (3) stability over time to ensure reproducibility, (4) easiness to obtain the desired shape according to the organ mimicked, (5) non-hazardousness, (6) easiness to prepare (7) cost, (8) minimal solidity to be manipulated. However, the presented phantom has some limitations regarding the anatomical fidelity and its dielectric properties compared to human tissues. Moreover, its modularity and flexibility could be further improved by 3D printed some elements with conductive filaments. Nonetheless, it has proved to be able to record a significant drop of resistance, matching the magnitude range of the human one when the bladder was filled. Consequently, the bladder fullness can be theoretically predicted from the magnitude of the resistance variation. Many challenges are still to be overcome, as the human body is far more complex than the proposed lab bench. In future work, underwear incorporating textile electrodes will be designed, manufactured, and tested on the developed lab bench. This smart underwear should be able to monitor the bladder fullness of its wearer to improve significantly its quality of life. 

## Figures and Tables

**Table 1:** Electrical conductivity values of the different tissues to be mimicked.

Tissue Name	Conductivity (S/m)	Percentage (%)	Weighted Conductivity
Fat	4.28 × 10^−2^	30	1.28 × 10^−2^
Colon/Rectum	2.38 × 10^−1^	5	1.19 × 10^−2^
Muscle	3.37 × 10^−1^	50	1.69 × 10^−1^
Bone marrow red	1.02 × 10^−1^	9	9.18 × 10^−3^
Bone marrow yellow	3.08 × 10^−3^	6	1.85 × 10^−4^
Spinal cord	3.46 × 10^−2^		
**Internal background**			**2.03** × 10^−1^
Urine	1.75		
Bladder wall	0.211		
Skin (dry)	0.000201		

**Table 2:** Content and electrical conductivity of the filling solutions.

Solution	Content	Conductivity
1	Deionized water	0 S/m
2	Background: Deionized water + NaCl (1g/L)	0.2 S/m
3	Deionized water + NaCl (5g/L)	1 S/m
4	Deionized water + NaCl (10g/L)	2 S/m
5	Deionized water + NaCl (30g/L)	6 S/m
